# Optimizing the Maximum Recovery of Dihydromyricetin from Chinese Vine Tea, *Ampelopsis grossedentata*, Using Response Surface Methodology

**DOI:** 10.3390/molecules22122250

**Published:** 2017-12-18

**Authors:** Umair Muhammad, Hedong Lu, Juan Wang, Jinzhi Han, Xiaoyu Zhu, Zhaoxin Lu, Sultana Tayyaba, Yousef I. Hassan

**Affiliations:** 1 College of Food Science and Technology, Nanjing Agricultural University, Nanjing 210095, China; umair_uaf@hotmail.com (M.U.); luhd100@163.com (H.L.); ha3341519@126.com (J.W.); hanjinzhi419@163.com (J.H.); zhuxiaoyu@njau.edu.cn (X.Z.); 2 College of Public Administration, Nanjing Agricultural University, Nanjing 210095, China; 2015209032@njau.edu.cn; 3 Guelph Research and Development Centre, Agriculture & Agri-Food Canada, 93 Stone Road West, Guelph, ON N1G 5C9, Canada; youhassan@yahoo.com

**Keywords:** dihydromyricetin, extraction, factors, temperature, solvent, time

## Abstract

This work provides an optimized extraction approach intended to maximize the recovery of dihydromyricetin (DHM) from Chinese vine tea (*Ampelopsis grossedentata*) leaves. The presented work adopts a Box-Behnken design as a response surface methodology to understand the role and influence of specific extraction parameters including: time, temperature, and solvent composition/ethanol (%) on DHM final yields. Initially, single factor experiments were used to delineate the role of above factors (temperature, time, and solvent composition) before proceeding with three factors-three levels Box-Behnken design with 17 separate runs to assess the effect of multifactorial treatments on DHM recovery rates. The collected data shows that independent variables (solvent composition, time, and temperature) can significantly affect DHM recovery rates with maximum yields resulting from a combined 60 °C, 60% aqueous ethanol, and 180 min treatment. From the empirical point of view, the above optimized extraction protocol can substantially enhance processing and profitability margins with a minimum need of interventions or associated costs.

## 1. Introduction

The utilization of plant-based products in natural medicine and health promotion can be traced back in time to the beginning of human civilization [1]. Plants were (and still are) considered as a paramount source of pharmacologically-active compounds with healing and health promoting characteristics attributable to the presence of secondary metabolites belonging to the phenolics, alkaloids, steroids, tannins, and flavonoids groups [2,3,4]. These secondary metabolites influence specific physiological/metabolic pathways in higher eukaryotes hence exhibiting their medicinal and therapeutic functionalities [5].

The diversity of the available botanical species coupled with the wealth of drugs and drug-precursors within these species makes investigating such natural sources a very lucrative theme despite the current and widely-spread preoccupation with synthetic chemistry as the dominant vehicle of discovering novel drugs/medications [6]. Some 75–80% of world’s population has a preference for using herbal medications and traditional therapeutics (comprised of plant extracts and plant-based active compounds) in comparison to synthetic drugs [7]. The recently reported advances in studying phytochemicals, identifying their health-promoting capabilities, and highlighting their effectiveness in addressing and preventing some chronic diseases (such as hypertension and *diabetes mellitus* type II) are expected to attract more attention to herbal remedies and encourage their use within the near future [8].

Moreover, medicinal plants are considered as important sources of economic value in many parts of the modern world. The production, preparation, and storage of raw plant materials in addition to the extraction processes of active ingredients are growing businesses in many oriental societies/cultures [9]. More recently, the above activities started to spread quite rapidly worldwide and are no more confined to one geographical area as new markets located within the western hemisphere are witnessing the fastest growing sales within the phytotherapy and evidence-based conventional medicine sectors [10,11].

**Figure 1:**
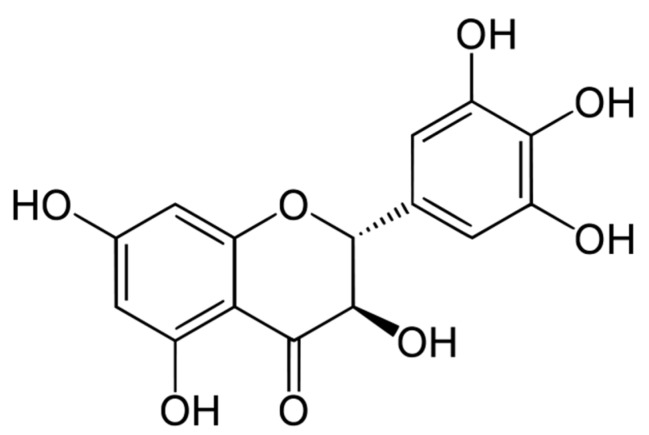
The chemical structure of dihydromyricetin (DHM): an active flavonoid [(2*R*,3*R*)-3,5,7-trihydroxy-2-(3,4,5-trihydroxyphenyl)-2,3-dihydrochromen-4-one, PubChem CID: 161557] found in abundance within *Ampelopsis grossedentata* leaves.

Dihydromyricetin (Figure 1), known also as ampelopsin, is a major secondary metabolite of the Chinese vine tea plant (*Ampelopsis grossedentata*) that belongs to the flavonoids category with reportedly strong anti-oxidant [12], anti-bacterial [13], anti-hypertension, anti-cancer [14], hepato-protective and anti-alcohol intoxication [15] properties. Due to the therapeutic values of dihydromyricetin (DHM), this compound is currently emerging as a promising bioactive ingredient intended for a number of pharmaceutical and functional food applications.

*Ampelopsis grossedentata* leaves are extremely rich in DHM, making this plant the preferred source of large-scale DHM production/extractions. While there are some preliminary studies that addressed the extraction of DHM from plant materials [16,17]; an optimized, simple, robust, and efficient large-scale extraction procedure is still desirable [16,17] in the light of the low-efficiency protocols that are currently practiced for DHM purification in many developing-countries.

The purpose of our presented work was to understand the experimental conditions (extraction temperatures, times and solvent composition) that influence DHM extractability from the widely spread Chinese vine tea leaves and to achieve maximum DHM yields without the need for laborious or highly specialized protocols/equipment. The reported optimized conditions should shed the light on the best conditions for increasing DHM yields hence facilitate the adoption/use of this natural aglycone flavonoid in a number of documented downstream health-promoting applications/functionalities.

## 2. Results and Discussion

### 2.1. Solvent Composition/Ethanol Percentage Affected DHM Extractability and Overall Yields

**Figure 2:**
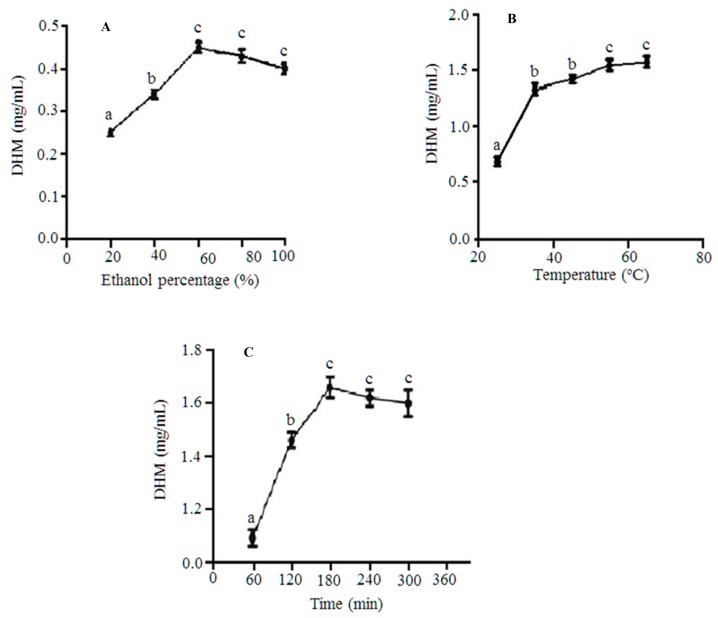
The effect of: (**A**) solvent composition/ethanol (%), (**B**) extraction temperature(s), and (**C**) extraction time(s) on DHM yields. Data are expressed as means ± standard deviations (SD) of three independent assays. Data points carrying different alphabetical designations represent statistically significant differences/observations (*p* < 0.05). Extraction variables (when not shown) were fixed at time = 150 min, temperature = 50 °C, and ethanol = 60%.

To evaluate the effect of solvent composition/ethanol (%) on DHM extractability, a wide-range of different concentrations of aqueous ethanol ranging from 20 to 100% was tested. Our results clearly showed that the ethanol content has a significant effect on DHM extraction/yields (Figure 2A). In essence, the overall yields of DHM increased as ethanol concentrations were increasing gradually up to 60%. After the 60% threshold, a negative correlation was evident between ethanol and the retrieved DHM (Figure 2A).

The simplest explanation for the above phenomenon, and for the increasing recovery of DHM as the percentage of water was decreasing within the extraction solvent, is the fact that “like dissolves like” as DHM was reported to have good solubility in methanol, ethanol, and acetone [18]. Based on the above observation, the identified optimum extraction point (60%) was chosen later as the starting point for the Box-Behnken design aiming at determining the optimal extraction time(s)/temperature(s) combination(s) with three levels of variation ranging from 55% to 65% (Table 1).

### 2.2. Increasing the Temperature of Extraction Mixtures Enhanced DHM Yields

To determine the most suitable temperature for DHM extraction from the Chinese vine tea, we tested a wide range of heating temperatures spanning the 25 to 65 °C range. The obtained results (Figure 2B) demonstrated a positive correlation between heating inputs and DHM yields while the temperature was increasing from room temperature reaching to 60 °C. A further increase to 65 °C did not show any additional positive effect. Our results in this regard are in agreement with Zhang et al. [19] who showed that heating (within 15–50 °C range) aided indeed in increasing DHM recovery rates.

The positive correlation between higher extraction temperatures and DHM yields is explained by the fact that higher temperatures facilitate the retrieval of many phenolic compounds through the enhancement of solvent diffusibility, improvement of phenolic compounds solubility [20,21,22], decreasing solvent viscosity, and reducing surface tensions [23]. Furthermore, mild heating improves the extraction of active compounds from plant materials by softening tissues and weakening the integrity of cellular walls [23,24]. These factors act together synergistically to increase the overall yields. Similar findings about the effect of heating on the yields of other phenolic compounds have been reported previously [20,25,26].

Based on our initial observations, the optimal extraction temperature was set at 60 °C while 55, 60 and 65 °C were chosen as the three experimental levels (lower, middle and upper) to proceed with the response surface approach.

### 2.3. Extending Extraction Times Positively Influenced DHM Overall Yields

To elucidate the influence of extraction times on DHM recoverability, we investigated five different time frames (60, 120, 180, 240, and 300 min), respectively. DHM recovery rates gradually increased through increasing the extraction times (60 min = 1.1 mg/mL, 120 min = 1.47 mg/mL, 180 min = 1.68 mg/mL, 240 min = 1.63 mg/mL, and 300 min = 1.59 mg/mL) with a maximum extractability around 180 min. However, extending the extraction duration beyond that point eliminated any positive correlation noted earlier between this factor and DHM yields and showed instead a negative impact (Figure 2C). This finding is in compliance with previously reported studies that observed a negative effect for prolonging the extraction time(s) beyond a certain threshold due to the increased risks of degradation of heat-sensitive phenolics [27,28]. Based on the above outcome, 180 min was chosen as the starting point for the second round of optimizations coupled with two other levels (170 and 190 min) respectively representing low, medium, and high values of this particular factor.

**Figure 3:**
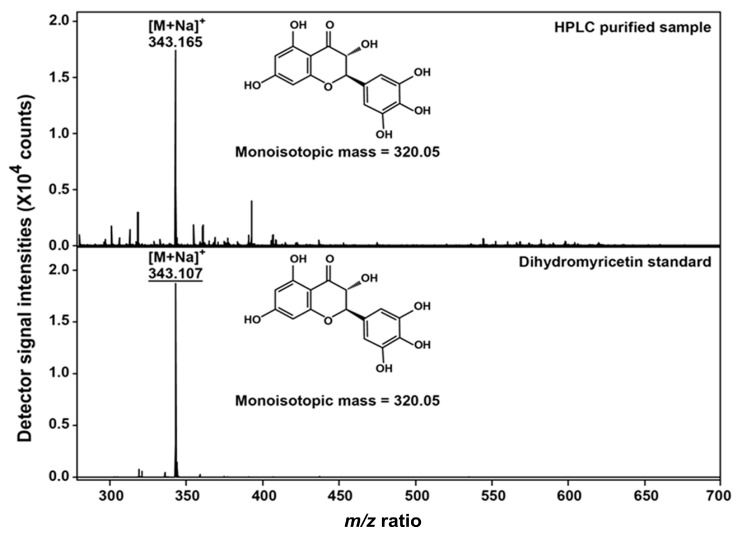
The obtained spectra of a HPLC-purified DHM sample (top) using matrix assisted laser desorption ionization-time of flight mass spectrometry (MALDI-TOF MS) in comparison to a DHM commercial standard spectra (bottom). Both the standard and sample were lyophilized and re-suspended in 10 µL H_2_O before mixing with 2,5-dihydroxybenzoic acid (DHB) solution [containing 4 mg/mL DHB in 30% acetonitrile, 0.1% TFA, and 1 mM sodium chloride] in 1:1 ratio before subsequent analysis.

As a confirmatory step, the identity of the purified compound was verified by the MALDI-TOF approach following the experimental approach detailed within the Material and Methods section. Figure 3 clearly shows that the obtained compound (with a molecular mass estimated at 320.05 Da) was indeed DHM that endured all the chemical characteristics/spectra of the commercially obtained DHM standards.

### 2.4. Using the Box-Behnken Design (BBD) to Optimize DHM Extractions and Testing Model Compliance with the Quadratic Fit

After establishing the preliminary effect of each extraction parameter (solvent composition/ethanol percentage, temperature and time) individually on DHM yields, the influence of these factors collectively on DHM recovery rates was delineated through a response surface methodology approach using the Box-Behnken Design (BBD).

We utilized three variables-three levels (low, middle, and high coded as −1, 0, and +1; respectively) and 17 different runs recording the hypothetical and experimental outcomes (DHM yields) for each of these combination treatments (Table 2).

Furthermore and to find the competency of the BBD-developed model and the significance of the associated factors, an analysis of variance (ANOVA) was performed on the obtained results. The outcomes are summarized in Table 3.

**Figure 4:**
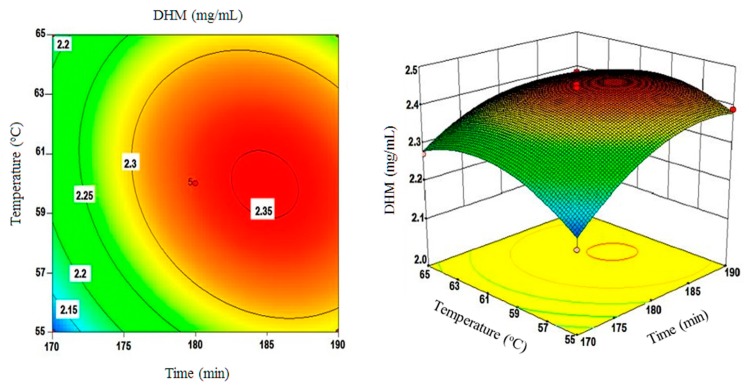
A three dimensional (3D) surface plot depicting the effect of extraction time(s) and extraction temperature(s) interactions on the yields of DHM (mg/mL).

**Figure 5:**
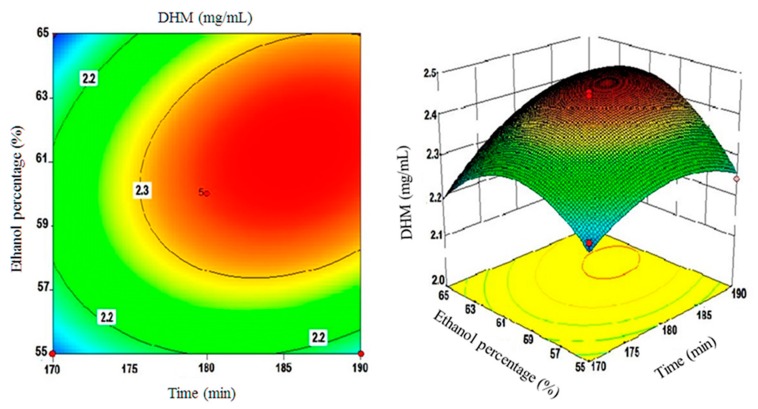
A 3D surface plot of ethanol (%) and extraction time(s) interactions and their effect on DHM yields (mg/mL).

**Figure 6:**
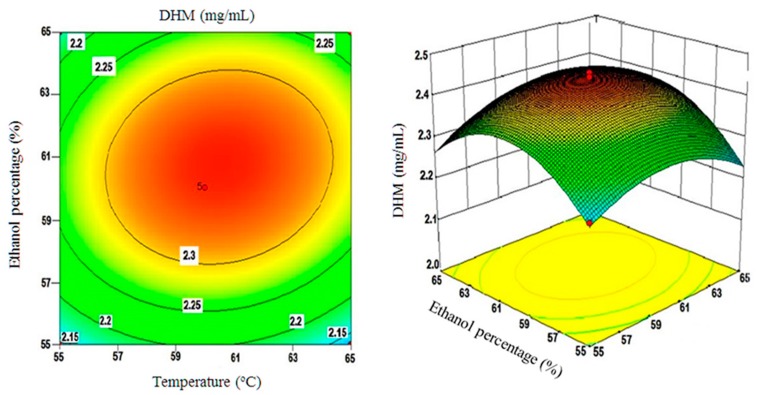
A 3D surface plot of ethanol (%) and extraction temperature(s) interactions and their effect on DHM yields (mg/mL).

Moreover, three-dimensional plots of surface responses were assembled by changing two variables within the experimental range and holding the third variable constant at the central point. These plots are depicted in Figure 4, Figure 5 and Figure 6.

The linear regression analysis that we have conducted and the significance levels (at *p* < 0.05) clearly reflected that the experimentally collected data adequately fitted the quadratic model. Based on the reported P-value, the significance of each variable coefficient was determined (Table 3) and the results indicated that A, C, AC, A^2^, B^2^, C^2^ were significant where A, B and C stand for the extraction time, temperature, and solvent composition/ethanol percentage, respectively.

Moreover, the results showed that the extraction time (designated as A) (*p* < 0.0004) was the most influential variable among all the independent factors followed by solvent composition (designated as C) (*p* = 0.0240) and finally the extraction temperature (designated as B) (*p* = 0.2934) (Table 3). In a like manner, extraction time(s) and solvent composition (AC) interactions were found to be statistically significant (*p* < 0.05) in their influence on DHM extraction yields (Table 3). However, the interactions of time and temperature (AB) or temperature and solvent composition (BC) were not enough significant to influence DHM yields (*p* > 0.05).

The F-value (=20.13) confirmed the reliability and significance of the developed model. The value of *R*^2^ (determination coefficient) was 0.96 while the “adj. *R*^2^” was 0.91 reflecting a high correlation between the experimentally obtained data and the predicted values (Table 3). Furthermore, the “lack of fit” value (=3.12) proved to be non-significant while the “coefficient of variance” was indeed low. Due to the above estimations, the model was considered reliable with a good distribution/coverage of data [29].

It is postulated that some non-accounted for yet non-significant variability could be embedded within any developed quadratic model and in order to reduce the influence/interference of such noise on the reported interactions between the tested experimental factors (time, temperature, and solvent in our case), we refined the predicted second order polynomial equation after deleting the non-significant variability according to following equation:

DHM = 2.34 + 0.062 × A + 0.028 × C + 0.041 × A × C − 0.064 × A^2^ − 0.068 × B^2^ − 0.11 × C^2^ (1)

The above equation shows that the ethanol content (%) and the extraction temperature both have positive inter-correlations while extraction temperatures have negative correlations with extraction times. Similarly, extraction times and the solvent composition show a significant effect on DHM overall yields.

Figure 4 shows the correlation between extraction times and extraction temperatures. In short, the obtained results showed that DHM yields were roughly within the 2.08 to 2.35 mg/mL range when the time and temperature varied from 170 to 180 min and 55 to 60 °C, respectively. A decline in DHM yields was only observed after increasing extraction times beyond the 190 min or extraction temperatures beyond 65 °C decreasing to 2.2 mg/mL as a result of such increases. The inter-correlations between extraction times and solvent composition/ethanol (%) are shown in Figure 5. Ethanol percentages between 55–60% and extraction times between 174–180 min showed a positive correlation with DHM yields (2.09–2.35 mg/mL).

However, a further increase in these two parameters rendered the correlation to the negative spectrum that was particularly evident with input values close to 185–190 min and ethanol percentages of 63.5–65%. The statistical analysis of the developed model clearly showed that extraction times intertwined with ethanol percentages to significantly influence DHM yields (*p* < 0.05).

Figure 6 shows the effects of solvent composition and extraction temperatures on the yields of DHM. In essence, when aqueous ethanol with percentages less than 55% was used with extraction temperatures below 55 °C; DHM yields were minimal. By increasing extraction temperatures to 60 °C and ethanol percentages to 56–60%, yields of DHM increased substantially from 2.10 to 2.33 mg/mL. This positive correlation between solvent temperature and its ethanol content was abrogated when temperature exceeded 60 °C or the ethanol content surpassed the 60% level decreasing DHM yields below the 2.15 mg/mL threshold as noticed in Figure 6.

In short, this study is among the first to explore the optimization of DHM extraction(s) by the RSM approach and to report the optimal range(s) of extraction parameters (time, temperature, solvent composition/ethanol percentages) which were determined empirically by single factor experiments and an established BBD experimental approach with 17 separate runs. The results were found to fit the second-order polynomial regression model with C.V. 1.26% (Table 3). While the above variables showed a significant effect when tested individually on DHM yields as the case of temperature (with a maximum efficiency at 55–60 °C), or solvent composition (with a maximum efficiency at 60%), or the extraction period (with a maximum efficiency at 180 min); our results indicated that the magnitude of these individual factors influence on DHM yields can be organized in the following order: extraction times > the ethanol content (%) > extraction temperatures. The overall results demonstrated that water and ethanol in different proportions/mixes significantly affected the overall yield of DHM. Higher recovery rates of dihydromyricetin were obtained through the use of aqueous ethanol which surpassed the control treatment (water only as an extraction solvent). This is explained in turn by the polar nature of many polyphenols (including DHM) hence the suitability of organic solvents for their extraction. Furthermore and in all the above reported experimental combinations, its seems that heat initially promoted DHM extractability through increasing energy transfer and influencing solvent/DHM mixability yet after a certain threshold, DHM levels tended to decrease due to the possible degradation of many bioactive compounds under elevated temperatures including DHM. Our results in this regard are in agreement with previously reported studies [30].

### 2.5. The Experimental Validation of Optimal Extraction Conditions/Parameters

By using the Design Expert software package, we predicted the optimal parameters for DHM extractions from Chinese vine tea, *Ampelopsis grossedentata*, leaves which were as the following: ethanol percentage = 60%, extraction period = 180 min with an extraction temperature at 60 °C. These optimal extraction conditions were incorporated within multiple independent assays according to the procedure described earlier and DHM yields were tracked to validate the developed extraction model and determine both its reliability and accuracy. The collected data (Table 4) showed that the actual DHM recovery rates (2.33 mg/mL) obtained through three independent assays fall within the range of DHM values (2.31 mg/mL) predicted by utilizing our developed model without any significant deviation under the proposed optimal conditions with a relative error (RE) value estimated at 0.87%.

## 3. Materials and Methods

### 3.1. Plant Materials and Chemical Reagents

Fresh and dried *Ampelopsis grossedentata* leaves (5 kg) were purchased from a Chinese medical herbs/plants supplier (Guizhou Miaoyao Biotechnology Co. Ltd., Tongren, Guizhou, China). A dihydromyricetin [(2*R*,3*R*)-3,5,7-trihydroxy-2-(3,4,5-trihydroxyphenyl)-2,3-dihydrochromen-4-one] standard (Cat. # D101549, ≥98% purity) was obtained from Aladdin (Shanghai, China) while trifluoroacetic acid was purchased from TEDIA (Fairfield, OH, USA). Methanol (≥99.8% purity) and acetonitrile (HPLC-grade) used in HPLC mobile-phase preparation were both bought from Sinopharm Chemical Reagents (Ningbo, China). All other chemicals used in this study were of analytical-grade and were dissolved in Milli-Q water (Millipore Corporation, Burlington, MA, USA) before use.

### 3.2. Sample Preparation

Conical flasks (100 mL) each containing three grams of vine tea (dry powder) were extracted with 50 mL of solvent (water or aqueous ethanol). Flasks were sealed with Parafilm (Shanghai Suolaibao Biotechnology, Shanghai, China) and aluminum foils (Xindi Paper Model of Products, Wuxi, China) to minimize light exposure. Mixtures were kept at 150 rpm in a covered temperature-controlled water bath/shaker (Shanghai Enxin Co. Ltd., Shanghai, China) during the entire extraction process. All the extracts were collected and concentered using a rotary evaporator RE-5299 rotary evaporator (Shanghaiyarong, Shanghai, China) Extraction times, temperatures, and ethanol concentrations (and combinations of these factors) are reported somewhere else in the manuscript and where selected according to the optimized experimental design/strategy as described above. Ultimately, vine tea extracts were filtered through Whatman No. 1 filter papers (Cat. # GB/T1914-2007, Whatman International, Maidstone, UK) into amber vials for immediate downstream analyses without any further storage. The above extractions were carried out in triplicates.

### 3.3. DHM Analysis and Quantification by High Performance Liquid Chromatography (HPLC)

A 1260 Infinity series HPLC (Agilent Technologies, Richmond, VA, USA) equipped with an Agilent Porshell HC-C18 column (Cat. # 518905-902, 5 µm 4.6 × 250 mm) was used to analyze DHM. The flow rate was set at 0.6 mL/min using a binary mobile-phase composed of methanol/water [32:68 (*v*/*v*)] with the use of 0.1% trifluoroacetic acid (TFA) as a solvent modifier. The DAD detector (Agilent) was programed to scan the entire UV range (190 to 400 nm) whereas the 291 nm wavelength was used for DHM detection. The heights of detected peaks were used for DHM quantification after establishing a standard analytical-curve using the commercially obtained DHM.

### 3.4. Confirmatory Analysis of DHM Using Matrix Assisted Laser Desorption Ionization-Time of Flight (MALDI-TOF) Mass Spectrometry

A further analysis was conducted to confirm the identity of the isolated compound (DHM in this case) using matrix assisted laser desorption ionization-time of flight (MALDI-TOF) mass spectrometry (Autoflex Speed MALDI-TOF, Bruker Daltonics, Bremen, Germany). In short, 3 g of the ground leaves of Chinese vine tea were extracted with 60 mL of pure water at 80 °C and continuous stirring at 150 rpm for 40 min. After this initial extraction, the sample was filtered through a filter paper (with pore size = 5 µm) and the filtrate was partitioned with water/chloroform (50:50 *v*/*v*) and the aqueous phase was collected while the impurities associated with the chloroform phase were discarded. A second partitioning took place using water/ethyl acetate (50:50 *v*/*v*) and functional compounds (including DHM) dissolved into the ethyl acetate layer were retrieved for a concentration step using a rotatory evaporator (RE-5299, Shanghaiyarong, Shanghai, China) attached to a low-temperature cooling liquid circulating pump (CCA-20, Gongyi Yuhua Instrument Co. Ltd., Zhengzhou, China). These compounds were subjected in turn to a HPLC-purification step using similar conditions to the ones reported in Section 3.3. and the respective peak that corresponds to DHM (based on the retention time of DHM commercial standards) was collected. Upon the collection of enough DHM, the confirmatory analyses were pursued as mentioned below.

The retrieved sample was lyophilized and re-suspended in 10 µL H_2_O before mixing with the 2,5-dihydroxybenzoic acid (DHB) buffer [composed of 30% of 20 mg/mL DHB in acetonitrile and 70% aqueous phase of 0.1% TFA coupled with 1 mM sodium chloride] in 1:1 ratios. One microliter of the resulting mixture was spotted on the MTP 384 polished steel plate. The sample was allowed to air-dry before loading into the mass spectrometer. Mass spectra were obtained in the positive reflectron ion mode with an acceleration voltage of 19 kV and laser frequency of 1 Hz. The laser power was set at 40% to 100% of the maximum potential. Signals from 500 shots were accumulated for each spectrum. For the external calibration, a standard peptide mixture “Peptide Mix II” (supplied by Bruker Daltonics) was used.

### 3.5. Delineating the Influence of Single Factors on DHM Extraction/Recovery Rates and Establishing Variability Ranges

In any feasible extraction process, the choice of solvent(s), extraction time(s), and temperature(s) are considered fundamental and can critically influence the successful outcome of such process based on the physical, chemical, and functional properties of the extracted ingredients/compounds [31]. Traditional therapeutics/active compounds are generally water-soluble hence water (as a polar solvent) is the preferred primary medium during the extraction of such functional compounds. Despite the above fact, our preliminary findings (data not shown) indicated that a binary mixture of solvents (mainly ethanol and water) used for the extraction of DHM might give much better outcomes than monophasic solvents (water or ethanol used separately). Earlier studies have also demonstrated similar findings for the extraction of other medicinally active components with reportedly some higher yields when organic-solvents were incorporated [24,32].

To elaborate on the above theme and during the first phase of the presented research, we explored the effect of single factors including solvent composition/ethanol (%), extraction time(s), and extraction temperature(s) on the overall recovery of DHM (as the response variable) when extracted from commercial Chinese vine tea leaves. After evaluating the effect(s) of above three independent variables individually and establishing the initial ranges of performance for each parameter, we proceeded with a further optimization step of the DHM extraction/purification process using multifactorial treatments that incorporated combinations of the above factors and reported as described below:

#### 3.5.1. The Influence of Solvent Composition/Ethanol (%) on DHM Recovery

Initially, five different mixtures with varying composition/ethanol percentages were tested. Mixture of ethanol: water (*v*/*v*) were prepared at 20, 40, 60, 80, and 100% and used for DHM extraction as described above. These independent extractions were performed in triplicates and the obtained data correlating with the most promising range for DHM yields was further chosen to be included within the second-phase multifactorial runs [with optimized extraction time(s) and temperature(s)].

#### 3.5.2. Extraction Times

Increasing extraction time(s) spanning 60, 120, 180, 240, and 300 min were tested to evaluate the effect of this parameter on DHM yields and select the optimal time range for a maximum response analysis. The data was collected from three independent assays conducted separately.

#### 3.5.3. Temperature of Extraction

After elucidating the most efficient extraction time(s) and the optimal range of solvent composition/ethanol (%) for maximum DHM recovery rates, samples were subjected to 25, 35, 45, 55, and 65 °C extraction schemes to correlate temperature(s) with DHM extractability. The provided data was collected from three independent assays.

### 3.6. Optimizing DHM Extraction Parameters through Response Surface Methodology (RSM) and Model Fitting

After investigating the impact of each single variable on DHM extractability from *Ampelopsis grossedentata*, a full factorial Box-Behnken Design (BBD) was implemented (with three factors and three levels) (Table 1) to optimize the overall DHM extraction process. *N* is the number of experiments required for the BBD and is given by the following equation:

*N* = 2*K* (*K* − 1) + *C*_0_ (2)

where *K* represents the number of factors and C_0_ represent center-points values where −1 (low), 0 (middle) and 1 (high) represent codes for factors levels and these codes were calculated according to Equation (3) [33]:

(3) $$\frac{\left(X_{i}-X_{0}\right)}{\Delta X}=x_{i}$$

where $x_{i}$ the coded value of the variable is *X_i_* and *X*_0_ is the value of *X* at the center point and ΔX is the step change.

The RSM approach was used to determine the arrangement of independent variables and categorize them into three (low, center, high) levels as mentioned above while the response factor (*Y*) was determined in three independent assays and mean-values were used later for regression analysis. The experimental approach that was conducted is illustrated in Table 2. The optimal extraction conditions were determined by testing the empirical second order polynomial regression model. The quadratic model equation was assumed and the results were found to fit mathematically with model Equation (4):

(4) $$Y=\;B_{0}+\overset{k}{\underset{i=1}{\sum }}B_{i}X_{i}+\;\overset{k}{\underset{i=1}{\sum }}B_{ii}X_{i}{}^{2}+\;\overset{k}{\underset{i\neq j=1}{\sum }}B_{ij}X_{i\;}X_{j}\;+E\;$$

where *Y* was the predicted response (yield of DHM in mg/mL) and *X_i_* and *X_j_* represented the independent variables. The number of tested variables was set to *k* = 3. Regression coefficients were denoted by $B_{0}$ and $B_{i}$, $B_{ii}\;\mathrm{and}\;B_{ij}$ representing linear, quadratic and interaction (cross product) regression coefficients (Table 3).

The Design Expert software package (version 7.0, Stat-Ease Inc., Minneapolis, MN, USA) was used for the analysis of variance (ANOVA), calculation of regression coefficients, model fitting to the quadratic model of the response factor (*Y*) by the mean of coefficient of determination (*R*^2^). The significance of all terms was analyzed statistically at (*p* < 0.05).

### 3.7. Empirical Validation of the Established Model

To test the validity of the established model, extraction parameters (inputs) and DHM yields (outputs) were validated empirically against the hypothetical values calculated by RSM. Wet-bench extractions were conducted in triplicates using the optimized extraction parameters as reported above and the obtained DHM values were statistically compared with the values predicted by the RSM model to determine the relative error (RE) value.

## 4. Conclusions

This report is among the first to look into optimizing the extraction processes of dihydromyricetin (DHM), a medicinally-active compound used commonly in traditional medicine, from the leaves of the Chinese vine tea (*Ampelopsis grossedentata*). We successfully utilized a response surface methodology through a Box-Behnken Design to investigate three pivotal parameters that influence DHM extractions: solvent composition/ethanol percentages, temperatures, and extraction times [34]. Our optimized model was empirically validated in a later stage and was found to accurately predict actual DHM yields verified through conducting laboratory-scale extractions that were run separately. The collected results highlighted the importance of understanding how the above factors intertwined to influence total DHM yields and how the extraction process can be optimized by implementing approaches that maximize the recovery of such valuable end-product of future commercial extractions.

In essence, the above results clearly demonstrate the effectiveness of using combinatorial approaches in order to optimize and enhance the preparations/purifications of functional medicinal compounds (such as DHM) and the possibility of manipulating simple yet essential factors that significantly influence the recovery rates of such compounds during commercial applications.

## Figures and Tables

**Table 1:** Symbols and levels of independent-variables adopted in the developed Box-Behnken Design (BBD) for DHM extraction.

Factors	−1	0	1
X1: extraction time (min)	170	180	190
X2: extraction temperature (°C)	55	60	65
X3: solvent composition/ethanol (%)	55	60	65

**Table 2:** The predicted values of Box-Behnken Design (BBD) alongside the obtained responses expressed as the actual concentrations of DHM (mg/mL).

Run	*X1* (Time/min)	*X2* (Temp./°C)	*X3* (Ethanol/%)	Response Values (Y) as DHM (mg/mL)	Predicted Value	Residual
1	0 (180)	−1 (55)	1 (65)	2.305 ± 0.29	2.230	0.08
2	−1 (170)	1 (65)	0 (60)	2.172 ± 0.32	2.146	0.03
3	1 (190)	−1 (55)	0 (60)	2.142 ± 0.16	2.270	−0.13
4	1 (190)	0 (60)	1 (65)	2.355 ± 0.22	2.297	0.06
5	0 (180)	0 (60)	0 (60)	2.352 ± 0.32	2.340	0.01
6	−1 (170)	0 (60)	1 (65)	2.210 ± 0.11	2.091	0.12
7	−1 (170)	0 (60)	−1 (55)	2.290 ± 0.28	2.117	0.17
8	0 (180)	1 (65)	−1 (55)	2.117 ± 0.21	2.134	−0.02
9	0 (180)	−1 (55)	−1 (55)	2.345 ± 0.13	2.234	0.11
10	0 (180)	0 (60)	0 (60)	2.110 ± 0.23	2.340	−0.23
11	0 (180)	0 (60)	0 (60)	2.277 ± 0.19	2.340	−0.06
12	0 (180)	1 (65)	1 (65)	2.335 ± 0.25	2.240	0.09
13	0 (180)	0 (60)	0 (60)	2.285 ± 0.22	2.340	−0.05
14	−1 (170)	−1 (55)	0 (60)	2.080 ± 0.14	2.146	−0.07
15	0 (180)	0 (60)	0 (60)	2.147 ± 0.18	2.340	−0.19
16	1 (190)	0 (60)	−1 (55)	2.140 ± 0.22	2.159	−0.02
17	1 (190)	1 (65)	0 (60)	2.177 ± 0.32	2.270	−0.09

**Table 3:** The analysis of variance (ANOVA) of independent-variables influence on DHM yields using the developed quadratic surface response model and testing the significance of obtained regression coefficients.

Parameters	Response			
	Estimated Coefficients	F-Value	Prob > F	Status
Intercept				
Model		20.13	0.0003	Significant
Linear effect				
A (time)	0.062	38.63	0.0004	Significant
B (temperature)	0.011	1.29	0.2934	NS**
C (solvent)	0.028	8.24	0.0240	Significant
Interactive effect				
AB	−0.024	3.03	0.1254	NS
AC	0.041	8.67	0.0216	Significant
BC	0.016	1.24	0.3015	NS
Quadratic effect				
A^2^	−0.064	21.72	0.0023	Significant
B^2^	−0.068	24.81	0.0016	Significant
C^2^	−0.11	61.86	0.0001	Significant
**The statistical parameters for fitting analysis**				
Lack of Fit	-	3.12	0.1503	NS
*R*^2^	-	-	0.9600	-
Adj. *R*^2^	-	-	0.9100	-
C.V. %	-	-	1.2600	-
Adequate precision	-	-	11.420	-

NS** = Not significant.

**Table 4:** The experimental validation of optimal extraction conditions of DHM from Chinese vine tea, *Ampelopsis grossedentata*, leaves as reported by this study. The predicted and actual yields of DHM obtained either through the developed BBD model or empirically through wet-extractions are shown.

	Extraction Times (min)	Extraction Temp. (°C)	Ethanol (%)	DHM (mg/mL)
Predicated	180	60	60	2.33
Experimental	180	60	60	2.31
RE (%)	-	-	-	0.87
